# Supplementary material for: T Cell Peptide Prediction, Immune Response, and Host–Pathogen Relationship in Vaccinated and Recovered from Mild COVID-19 Subjects
Source: Biomolecules. 2024 Sep 26;14(10):1217. doi: 10.3390/biom14101217 (PMC11505848; doi:10.3390/biom14101217)
Supplement: Supplementary file 1 [file biomolecules-14-01217-s001.zip › biomolecules-3156215-supplementary.pdf]

T cell peptide prediction, immune response, and host-pathogen relationship in vaccinated and recovered from mild COVID-19 subjects

Iole Macchia[1#], Valentina La Sorsa[2#], Alessandra Ciervo[3], Irene Ruspantini[4], Donatella Negri[3], Martina Borghi[3], Maria Laura De Angelis[1], Francesca Luciani[5], Antonio Martina[5], Silvia Taglieri[1], Valentina Durastanti[6], Maria Concetta Altavista[6], Fabiola Mancini[3†] and Francesca Urbani[1†§]

Supplementary material:

- VOC mutated 9-11mer peptide details
- Long peptide details
- Supplementary tables
- Supplementary figures

VOC mutated 9-11mer peptide details

NL-9o

The Omicron derived Spike KL-9o_NIADYNYKL shared by Beta (QRN78347.1), Delta plus (Delta+), BA.1, BA.2, BA.4 and BA.5 variants, corresponded to the Wuhan KL-9 epitope (found also in Delta and Alpha VOCs), and presented the mutation K417N known to reduce or even abolish binding to the monoclonal antibody ab1 (IgG1 ab1) (79). This substitution results in escape from class I neutralizing antibodies such as the therapeutic monoclonal antibody (MAb) etesevimab (80).

VV-9o

VG-9 Omicron peptide was also included in the study mainly for the presence of the substitution A67V presents in BA.1 strain and Δ69-70 found in most of Omicron [BA.1, BA.4 (UPP14409.1), BA.5 (UOZ45804.1)] and Alpha (QWE88920.1) VOCs. Notably, the deletion does not appear to contribute to antibody escape, although *in vitro* experiments show that it increases infectivity (81).

KA-10d

Similarly, we considered the KA-10δ peptide that carries the D950N mutation, although its NetMHCpan 4.1b % Rank was higher (1.841), if compared with its Wuhan counterpart. This mutation slightly promotes alone or in association with S-P681R, respectively, membrane fusion, Spike cleavage and fusogenicity (82).

VV-11od

The last mutant epitope selected, VV-11oδ _ VLYQGVNCTEV (residue 610), shared by Omicron BA.1, BA.2, BA.4, BA.5, Alfa, Beta, Gamma, and Delta variants, contains the D614G mutation. This mutation leads to a decrease in the % Rank and thus makes the peptide potentially more immunogenic. If presents this substitution, S1 dissociates with more difficulty than D614, suggesting that the G614 variant is more stable than the wild type and potentially, the virus more infectious (83). High nasopharyngeal viral RNA loads were found-in patients carrying this mutation, indicating a probable fitness advantage (84). Plante et al, conducted *in vitro* studies using different types of cells including human tracheal and bronchial epithelial in layers simulating the epithelial tissue of the respiratory tract, and observed that the D614G mutation increases the viral replication infectivity of SARS-CoV-2 produced from a human lung cell line (85).

References

79. Mannar D, Saville JW, Zhu X, Srivastava SS, Berezuk AM, Zhou S, et al. Structural analysis of receptor binding domain mutations in SARS-CoV-2 variants of concern that modulate ACE2 and antibody binding. Cell Rep [Internet]. 2021 Dec 21 [cited 2023 Jun 13];37(12). Available from: https://pubmed.ncbi.nlm.nih.gov/34914928/

80. Laurini E, Marson D, Aulic S, Fermeglia A, Pricl S. Molecular rationale for SARS-CoV-2 spike circulating mutations able to escape bamlanivimab and etesevimab monoclonal antibodies. Sci Rep [Internet]. 2021;11(1):1–20. Available from: https://doi.org/10.1038/s41598-021-99827-3

81. Meng B, Kemp SA, Papa G, Datir R, Ferreira IATM, Marelli S, et al. Recurrent emergence of SARS-CoV-2 spike deletion H69/V70 and its role in the Alpha variant B.1.1.7. Cell Rep [Internet]. 2021 Jun 29 [cited 2023 Jun 13];35(13). Available from: https://pubmed.ncbi.nlm.nih.gov/34166617/

82. Furusawa Y, Kiso M, Iida S, Uraki R, Hirata Y, Imai M, et al. In SARS-CoV-2 delta variants, Spike-P681R and D950N promote membrane fusion, Spike-P681R enhances spike cleavage, but neither substitution affects pathogenicity in hamsters. eBioMedicine [Internet]. 2023;91:104561. Available from: https://doi.org/10.1016/j.ebiom.2023.104561

83. Zhang L, Jackson CB, Mou H, Ojha A, Peng H, Quinlan BD, et al. SARS-CoV-2 spike-protein D614G mutation increases virion spike density and infectivity. Nat Commun. 2020;11(1):1–9.

84. Korber B, Fischer WM, Gnanakaran S, Yoon H, Theiler J, Abfalterer W, et al. Tracking Changes in SARS-CoV-2 Spike: Evidence that D614G Increases Infectivity of the COVID-19 Virus. Cell [Internet]. 2020 Aug 20 [cited 2023 Jun 13];182(4):812-827.e19. Available from: https://pubmed.ncbi.nlm.nih.gov/32697968/

85. Plante JA, Liu Y, Liu J, Xia H, Johnson BA, Lokugamage KG, et al. Spike mutation D614G alters SARS-CoV-2 fitness. Nature [Internet]. 2021;592(7852):116–21. Available from: http://dx.doi.org/10.1038/s41586-020-2895-3

Long peptide details

Long peptide 135w/δ

Besides R158/del, LP 135δ carries G142D, E156G, and F157 mutations, which have been found during delta outbreaks and combined with the L452R mutation seem to have contributed to increased infectivity and reduced susceptibility to neutralization (121). A previous study by Chaudahari and colleagues, showed that the Delta variant carrying mutations (E156G, F157, R158/del) in the N-terminal domain (NTD) leads to immune evasion towards 4A8 monoclonal antibody directed against the NTD, both in molecular docking studies and in neutralization-based assays on pseudoviruses (122). Wuhan NTD is a beta-strand, and the E156G and F157, R158/del mutations remodel the structure by interrupting it with an alpha fold. An X-ray crystalline graphic study demonstrated that the presence of the alpha fold makes the virus less susceptible to binding to monoclonal antibodies (123).

Long peptide 203w/o

In a previous study, a reduced affinity was demonstrated for a Wuhan Spike protein peptide HLADRB1* 03:01 allele due to mutations in BA.1 (N211 deletion, L212I substitution, insert 212–214 EPE, identified in the N-terminal domain (NTD) in case of the BA.1 variant. The lower affinity bioinformatics prediction was confirmed by ELISA (124).

References

121. Mishra T, Joshi G, Kumar A, Dalavi R, Pandey P, Shukla S, et al. B.1.617.3 SARS CoV-2 spike E156G/Δ157-158 mutations contribute to reduced neutralization sensitivity and increased infectivity. bioRxiv [Internet]. 2021;2021.10.04.463028. Available from: https://www.biorxiv.org/content/10.1101/2021.10.04.463028v1%0Ahttps://www.biorxiv.org/content/10.1101/2021.10.04.463028v1.abstract

122. Chaudhari AM, Joshi M, Kumar D, Patel A, Lokhande KB, Krishnan A, et al. Evaluation of immune evasion in SARS-CoV-2 Delta and Omicron variants. Comput Struct Biotechnol J [Internet]. 2022;20:4501–16. Available from: https://doi.org/10.1016/j.csbj.2022.08.010

123. McCallum M, Walls AC, Sprouse KR, Bowen JE, Rosen LE, Dang H V., et al. Molecular basis of immune evasion by the Delta and Kappa SARS-CoV-2 variants. Science (80-). 2021;374(6575):1621–6.

124. Nersisyan S, Zhiyanov A, Zakharova M, Ishina I, Kurbatskaia I, Mamedov A, et al. Alterations in SARS-CoV-2 Omicron and Delta peptides presentation by HLA molecules. PeerJ. 2022;10:1–15.

Supplementary Table S1. Major epitopes of selected long peptides and their associated haplotype

ID	Sequence	Haplotype	T cell Epitope	%Rank
135w (32 aa)	FCNDPFLGVYYHKNNKSWMESEFRVYSSANNC	HLA-A*01:01	FCNDPFLGVY	0.381
			WMESEFRVY	0.382
			CNDPFLGVYY	0.385
			FCNDPFLGVYY	0.594
			CNDPFLGVY	0.394
		HLA-A*03:01	GVYYHKNNK	0.046
		HLA-A*24:02	YYHKNNKSW	0.081
			VYYHKNNKSW	0.199
		HLA-B*15:01	WMESEFRVY	0.492
135δ (30 aa)	FCNDPFLDVYYHKNNKSWMESΔΔGVYSSANNC	HLA-A*01:01	FCNDPFLDVY	0.249
			CNDPFLDVY	0.286
			KSWMESGVY	0.57
			CNDPFLDVYY	0.444
		HLA-A*24:02	YYHKNNKSW	0.081
			VYYHKNNKSW	0.199
		HLA-B*15:01	KSWMESGVY	0.483
203w (28 aa)	IYSKHTPINLVR---DLPQGFSALEPLVDLP	HLA-A*24:02	IYSKHTPINL	0.202
		HLA-A*26:01	LVRDLPQGF	0.364
		HLA-B*07:02	LPQGFSAL	0.225
			TPINLVRDL	0.336
		HLA-B*15:01	LVRDLPQGF	0.184
203o (31 aa)	IYSKHTPIDIVREPEDLPQGFSALEPLVDLP	HLA-A*24:02	IYSKHTPII	0.057
		HLA-B*07:02	LPQGFSAL	0.225
		HLA-B*40:01	REPEDLPQGF	0.462

Supplementary Table S2. Identity between SARS-CoV-2 and common human coronavirus 9-11mer peptides

			% of aa in common with SARS-CoV-2			
**Protein**	**ID**	**Conserved/mutated/present in Alpha, Beta, Gamma, Delta, BA.1, BA.2, BA.4, BA.5 VOCs**	**OC43**	**HKU1**	**NL63**	**229E**
Spike	KA10	Conserved in Alpha, Beta, Gamma VOCs	70	70	70	70
	KA10δ	Present in Delta VOC	70	70	60	60
	KL9	Conserved in Alpha and Delta VOCs	55	55	33	66
	NL9o	Present in Beta, BA.1, BA.2, BA.4, BA.5 VOCs	55	55	33	66
	VV11	Mutated in all VOCs	45	45	36	55
	VV11δo	Present in all VOCs	45	36	36	55
	VV9	Conserved in Beta, Gamma, Delta, BA.2 VOCs	11	11	44	11
	VG9o	Present in BA.1 VOC	55	11	44	44
	FV10	Conserved in all VOCs	40	50	40	40
	GL9	Conserved in all VOCs	77	66	44	66
	KV10	Conserved in all VOCs	60	70	80	80
	LA9	Conserved in all VOCs	66	77	66	55
	TL10	Conserved in all VOCs	50	50	40	40
	TL9	Conserved in all VOCs	66	44	55	44
	VA11	Conserved in all VOCs	55	55	45	45
	VI9	Conserved in all VOCs	77	77	66	66
	YL9	Conserved in all VOCs	66	11	44	44
Nsp1	QV9	Conserved in all VOCs	44	44	44	55
	TV9	Conserved in all VOCs	55	55	55	66
	VL9	Conserved in all VOCs	44	44	77	77
Nsp2	FV9	Conserved in all VOCs	33	44	22	33
	RT9	Conserved in all VOCs	44	44	44	44
	TI9	Conserved in all VOCs	44	33	44	44
Nsp3	FI10	Conserved in all VOCs	40	40	60	40
	IV9	Conserved in all VOCs	44	55	66	44
	KL10	Conserved in all VOCs, except in Alpha	40	60	40	30
Nsp16	QL9	Conserved in all VOCs	77	55	66	77
	SL10	Conserved in all VOCs	**90**	**90**	50	40
	WV9	Conserved in all VOCs	77	66	44	55

>70 % values are highlighted in yellow
in bold: probable cross-reaction between SARS-CoV-2 and common coronavirus

Supplementary Table S3 Symptoms.

Subj#	Cough	Headache	Fever	Rapid pulse	Confusional state	Throath pain	Abdominal pain	Rynorrhea	Vomiting nausea	Diarrhea
1	NO	NO	NO	YES	NO	NO	NO	NO	NO	NO
2	YES	NO	YES	NO	YES	NO	NO	NO	NO	YES
3	YES	YES	YES	YES	YES	YES	YES	NO	NO	NO
4	NO	YES	YES	NO	YES	NO	NO	NO	NO	NO
5	NO	YES	NO	NO	NO	NO	NO	NO	NO	NO
6	NO	NO	NO	NO	NO	NO	NO	YES	NO	NO
7	YES	YES	NO	NO	NO	YES	NO	NO	YES	YES
8	YES	NO	NO	NO	NO	YES	NO	NO	NO	NO
9	NO	NO	YES	NO	NO	NO	NO	YES	NO	NO
10	NO	YES	NO	NO	NO	YES	NO	NO	NO	NO
11	NO	NO	YES	NO	NO	NO	NO	YES	NO	NO
12	YES	NO	YES	NO	NO	YES	NO	YES	NO	NO
13	NO	YES	YES	NO	NO	YES	NO	NO	NO	NO
14	NO	YES	NO	NO	NO	YES	NO	YES	NO	NO

Subj#	Thoracic pain	Conjunctivitis	Muscle pain	Rush	Articular pain	Malaise-Fatigue	Ageusia	Anosmia	Shortness of breath	Other symptom
1	NO	NO	NO	NO	NO	NO	NO	NO	NO	NO
2	NO	NO	NO	NO	NO	YES	YES	YES	NO	NO
3	NO	NO	YES	NO	YES	YES	YES	YES	YES	NO
4	NO	NO	NO	NO	NO	YES	NO	NO	NO	NO
5	YES	NO	NO	NO	YES	NO	NO	NO	NO	NO
6	NO	YES	NO	NO	NO	NO	NO	NO	NO	NO
7	NO	NO	NO	YES	NO	NO	YES	NO	NO	NO
8	NO	NO	NO	NO	NO	YES	NO	NO	NO	NO
9	NO	NO	NO	NO	NO	NO	YES	YES	NO	NO
10	NO	YES	NO	NO	NO	YES	NO	NO	YES	NO
11	NO	NO	NO	NO	NO	NO	YES	YES	NO	YES
12	NO	NO	YES	NO	YES	YES	NO	NO	NO	NO
13	NO	NO	YES	NO	NO	YES	NO	NO	NO	NO
14	NO	NO	YES	NO	YES	YES	NO	NO	NO	YES

No subjects showed any of the subsequent symptoms: Inability to walk, Wheezing, Bleeding, Lymphadenopathy, Seizures, Lower chest indrawing, Skin ulcer

Supplementary Table S4. Other clinical/lyfestyle characteristics of enrolled subjects.

Subj#	Concomitant therapy	Paracetamol	Previous pathology	Previous infectious disease	Allergy	Flu vax	Alcohol consumption	Tobacco use	Intensive sport activity
1	YES	NO	YES	YES	NO	NO	NO	NO	NO
2	YES	NO	NO	NO	NO	NO	NO	NO	NO
3	YES	NO	NO	NO	NO	NO	NO	NO	NO
4	YES	YES	YES	YES	YES	NO	NO	NO	YES
5	NO	NO	NO	NO	NO	NO	NO	NO	YES
6	YES	YES	NO	NO	NO	YES	NO	NO	NO
7	YES	YES	NO	NO	NO	NO	YES	NO	YES
8	YES	YES	YES	NO	NO	NO	YES	NO	NO
9	YES	NO	NO	NO	NO	YES	NO	YES	NO
10	YES	NO	NO	NO	NO	NO	NO	NO	NO
11	NO	NO	NO	NO	NO	NO	YES	NO	NO
12	YES	NO	YES	YES	YES	NO	NO	NO	NO
13	NO	NO	NO	NO	NO	NO	NO	NO	NO
14	YES	NO	YES	NO	NO	YES	NO	NO	NO

Supplementary Table S5. 7-color Flow Cytometry panel composition for naïve/memory T cell immunophenotyping

Staining panel	Antigen/Ligand	Fluorochrome	Clone	Species	Manufacturer	Nationality	Format
Major CD3+ T cell and γδ T cell memory/naive subsets	CD4	FITC	13B8,2	mouse	Beckman Coulter	California	Dried
	CCR7 (CD197)	PE	G043H7				
	CD8	Pe Cy5.5	B9.11				
	CD3	Pe Cy7	UCHT-1				
	CD45RA	APC	2H4				
	Vd2 TCR	APC-Fire 750	B6		Biolegend		Liquid
	CD45	APC-Cy7	2D1		Beckman Coulter		

Supplementary Table S6. Parameters under study - demographic, clinical, lifestyle + T and B response variables.

	Demographic, clinical and lifestyle data / by Survey (52 variables)
	Sex at birth
	Age
	Blood collection/sampling date
	1st pos swab date
	1st neg swab date
	Last COVID-19 vaccine before blood collection date
	anti-SARS-CoV-2 vaccine manufacturer
	anti-SARS-CoV-2 vaccine dose number (1, 2 or 3)
	Fever
	Cough
	Headache
	Fast pulse rate
	Confusional state
	Sore throat
	Abdominal pain
	Rhinorrhea
	Vomiting/nausea
	Diarrhea
	Ageusia
	Anosmia
	Shortness of breath
	Chest pain
	Conjunctivitis
	Muscle pain
	SpO2 <96%
	Skin rash
	Joint pain
	Malaise-Fatigue
	Inability to walk
	Wheezing
	Bleeding
	Lymphadenopathy
	Seizures
	Lower chest indrawing
	Skin ulcer
	Other symptom
	Concomitant therapy
	Concomitant Paracetamol intake
	Previous pathology
	Previous infectious disease
	Allergy
	Tobacco use
	Alcohol consumption
	Flu vaccine
	Intensive sport activity
estimated/ calculated variables	Probable VOC (B 1.617.2 Delta, B.1.1.529 Omicron BA.1 or BA.2) infection
	Probable Delta / Omicron infection
	Time interval between 1st positive swab and sampling (PS-ΔT)
	Time interval between last vaccine dose and sampling (VS-ΔT)
	Time interval between last vaccine dose and 1st positive swab (VP-ΔT)
	Time interval between 1st positive swab and 1st negative swab (PN-ΔT)
	Total symptoms

T cell response / by Elispot (37 variables)
CEF
Peptivator N
Peptivator S
Spike 135d
Spike 135w
Spike 203O
Spike 203W
Spike KA-10d
Spike KA-10w
Spike KL-9w
Spike NL-9o
Spike VV-11od
Spike VV-11w
Spike VV-9w
Spike VG-9o
Spike FV-10
Spike GL-9
Spike KV-10
Spike LA-9
Spike TL-10
Spike TL-9
Spike VA-11
Spike VI-9
Spike YL-9
Nsp1 QV-9
Nsp1 TV-9
Nsp1 VL-9
Nsp2 FV-9
Nsp2 RT-9
Nsp2 TI-9
Nsp3 FI-10
Nsp3 IV-9
Nsp3 KL-10
Nsp16 QL-9
Nsp16 SL-10
Nsp16 WV-9
33-peptide response rate (calculated, see Table 3)

Plasma antibodies / by Elisa (2 variables)
anti-NP Abs
anti-Spike Abs

Neutralizing plasma anti-Spike antibodies / by pseudovirus *in house* test (6 variables)
anti-Wuhan nAbs
anti-Alpha nAbs
anti-Delta nAbs
anti-Omicron BA.1 nAbs
anti-Omicron BA.2 nAbs
anti-Omicron BA.4.5 nAbs

Supplementary Table S7. Parameters under study - MFC variables.

Major and $\gamma\delta$ TCR Vδ2 T lymphocyte subpopulation naïve-memory phenotype / by Multiparametric Flow Cytometry
(38 variables)

parent population	variable
Leukocytes	Lymphocytes
Lymphocytes	CD3
CD3	CD4sp
CD3	CD8sp
CD3	DP1
CD3	DP2
CD3	DN
CD3	Vδ2
CD3	N CD3
CD3	CM CD3
CD3	EM CD3
CD3	TD CD3
CD3	CD45RA CD3
CD3	CCR7 CD3
CD4	N CD4sp
CD4	CM CD4sp
CD4	EM CD4sp
CD4	TD CD4sp
CD8	N CD8sp
CD8	CM CD8sp
CD8	EM CD8sp
CD8	TD CD8sp
DP1	N DP1
DP1	CM DP1
DP1	EM DP1
DP1	TD DP1
DP2	N DP2
DP2	CM DP2
DP2	EM DP2
DP2	TD DP2
DN	N DN
DN	CM DN
DN	EM DN
DN	TD DN
Vδ2	Vδ2 N
Vδ2	Vδ2 CM
Vδ2	Vδ2 EM
Vδ2	Vδ2 TD

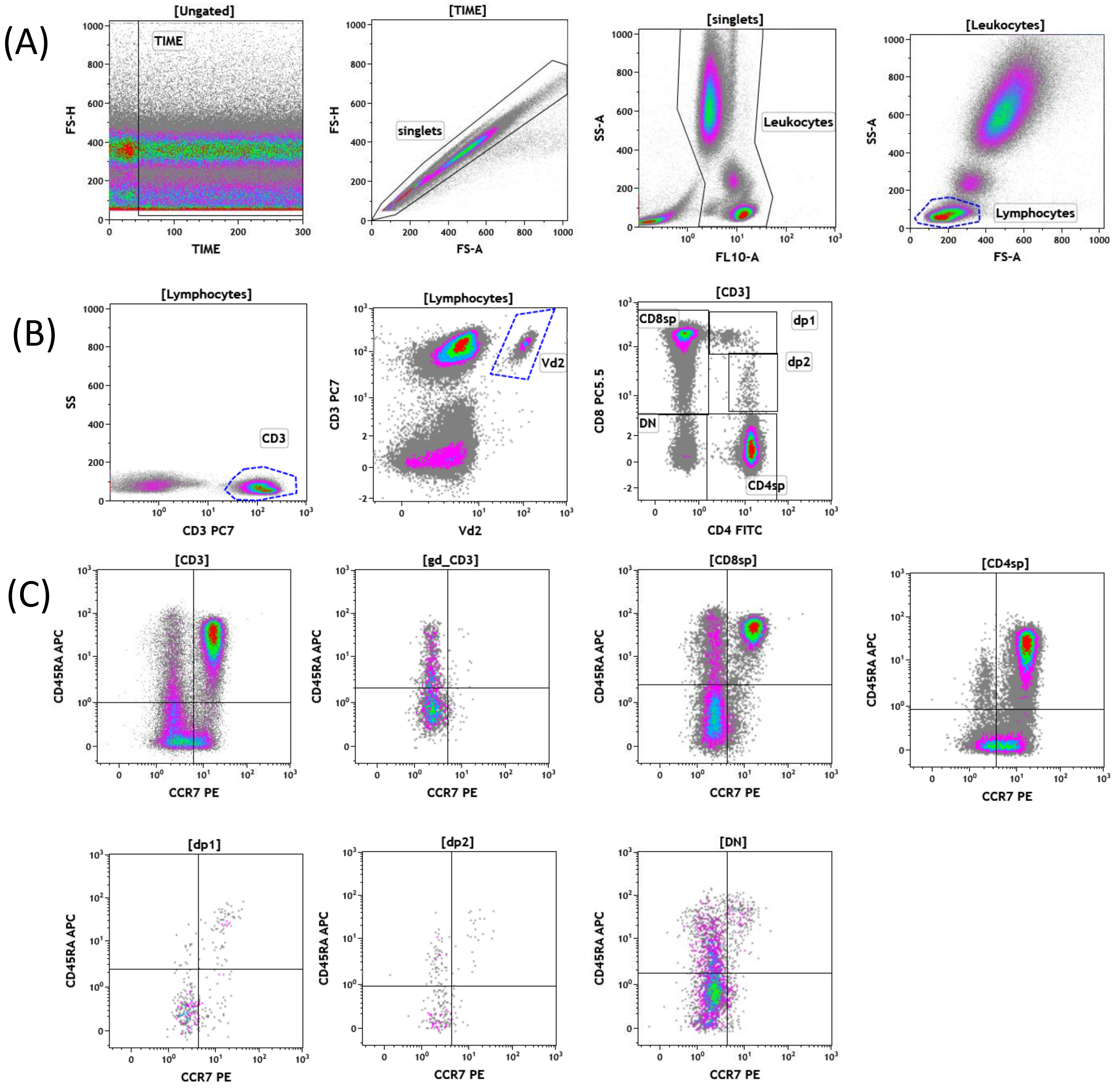

Supplementary Figure S1. Gating strategy for naïve/memory T cell panel. Representative dot plots depicting the gating strategy for analyzing different CD3$^+$ cell subsets. A, B and C) whole blood samples were stained with a six-color panel which includes anti-CD45RA, -Vδ2, -CCR7, -CD8, -CD4 and-CD3 mAbs. a) Unstable flow time-lapse, debris and cell aggregates were removed from the analysis by subsequently gating on TIME and singlet events (left and central plots). Leukocytes are defined as CD45+ cells. Lymphocytes were gated within a SSC-A/FSC-A dot plot (right plot). b) A CD3 *vs* TCR Vδ2 dot plot was drawn within the lymphocyte region, allowing the identification of total CD3$^+$ TCR Vδ2$^+$ (γδ) T cells (central plot). Single positive (either CD4$^+$ or CD8$^+$ T cells), double positive (dp) CD4$^+$CD8$^+$ (either CD8^{hi}CD4^{low} or CD8^{low}CD4^{hi}) T cells were identified within CD3$^+$ gated cells (right plot). c) The different T cell subsets were then distinguished in naïve (N, CD45RA$^+$ CCR7$^+$), central memory (CM, CD45RA$^-$CCR7$^+$), effector memory (EM, CD45RA$^-$CCR7$^-$), and terminally differentiated (TD, CD45RA$^+$CCR7$^-$) cells within each of the above-mentioned T cell subsets.

A

		netMHCpan 4.1b	netCTLpan
netCTLpan	rho	0.366	
	p	0.051	
	n	29	
Syfpeithi	rho	-0.506	-0.522
	p	0.008	0.006
	n	26	26

B

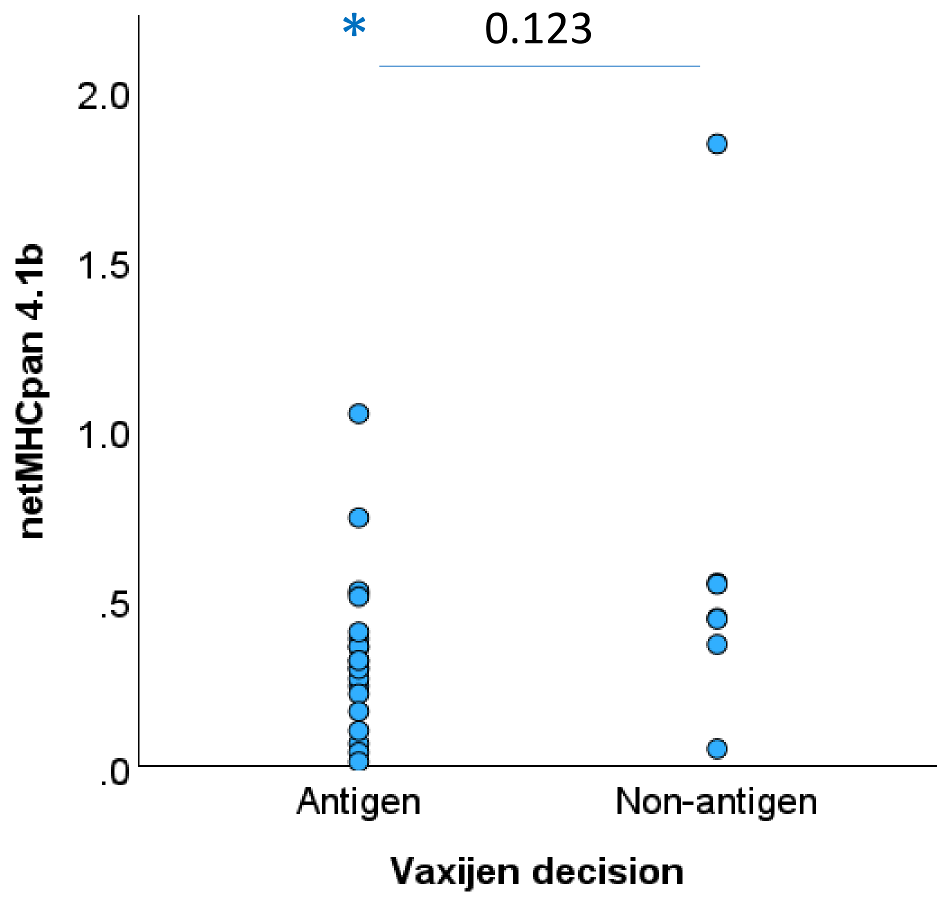

Supplementary Figure S2. Association between netMHCpan 4.1b % Rank and other algorithm scores. **A**) Spearman correlation with netCTLpan % Rank and Syfpeithi score. **B**) Scatter plot according to the Vaxijen decision (the *p*-value is calculated using the Mann-Whitney U test for independent variables). *: outlier.

Non-HLA*02:01 subjects' cPBMC ELISPOT results

A

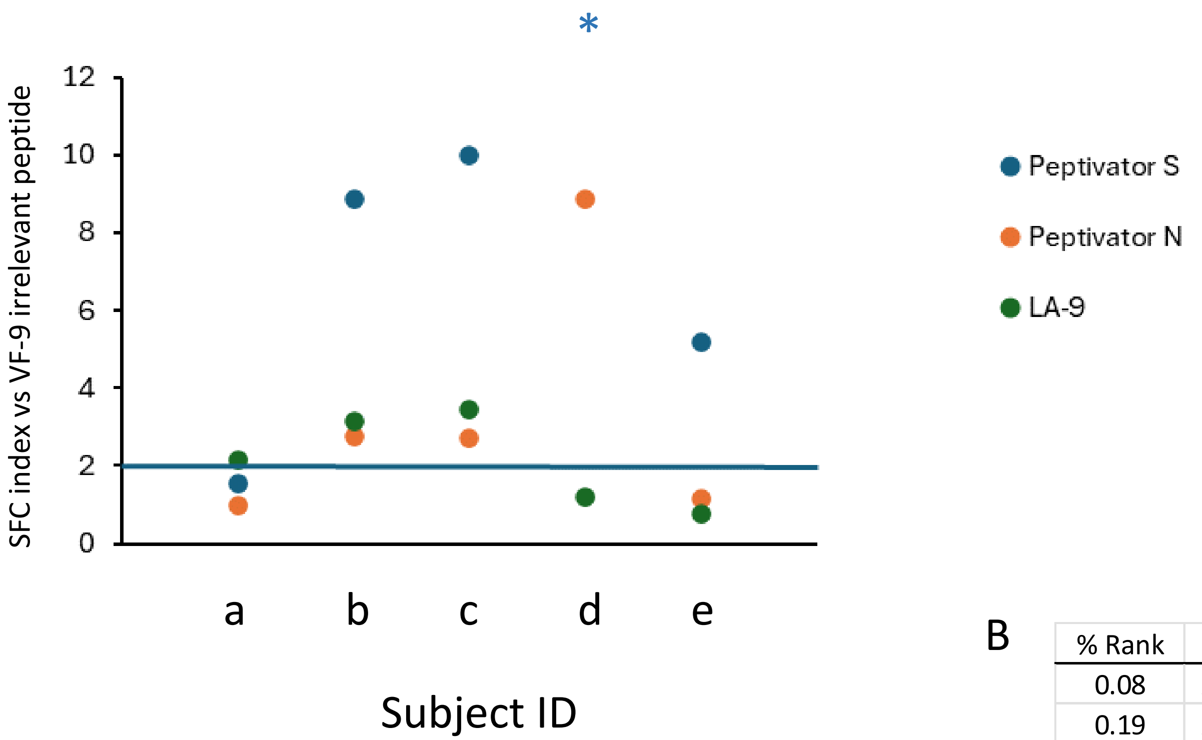

B

% Rank	Allele
0.08	A*02:01
0.19	A*02:06
0.08	B*08:01
0.92	B*13:02
0.92	B*14:02
0.93	B*48:01
0.86	B*52:01
1.12	B*53:01
0.90	C*01:02
1.50	C*03:03
1.50	C*03:04
1.60	C*07:04
1.60	C*16:01
1.10	C*17:01
0.56	E*01:03

Supplementary Figure S3. LA-9 as an immunogenic stimulator and binder to MHC-I molecules other than HLA-A*02:01. A) ELISPOT results of non-HLA-A*02:01 subjects' cPBMC stimulated with Peptivator S, Peptivator N and LA-9 peptide. The blue line indicates the arbitrary cut-off value (two-fold increment with respect to the VF-9 irrelevant peptide SFC value). **B**) List of the best % Rank values, calculated by NetMHCpan 4.1b, associated with the most common HLA alleles, generated by querying the IEDB using the LA-9 amino acid sequence. *: outlier value.

A

		netMHCpan 4.1b	netCTLpan	Syfpeithi
Immunogenicity rate	rho	-.440*	0.349	0.013
	p	0.025	0.081	0.949
	n	26	26	26

B

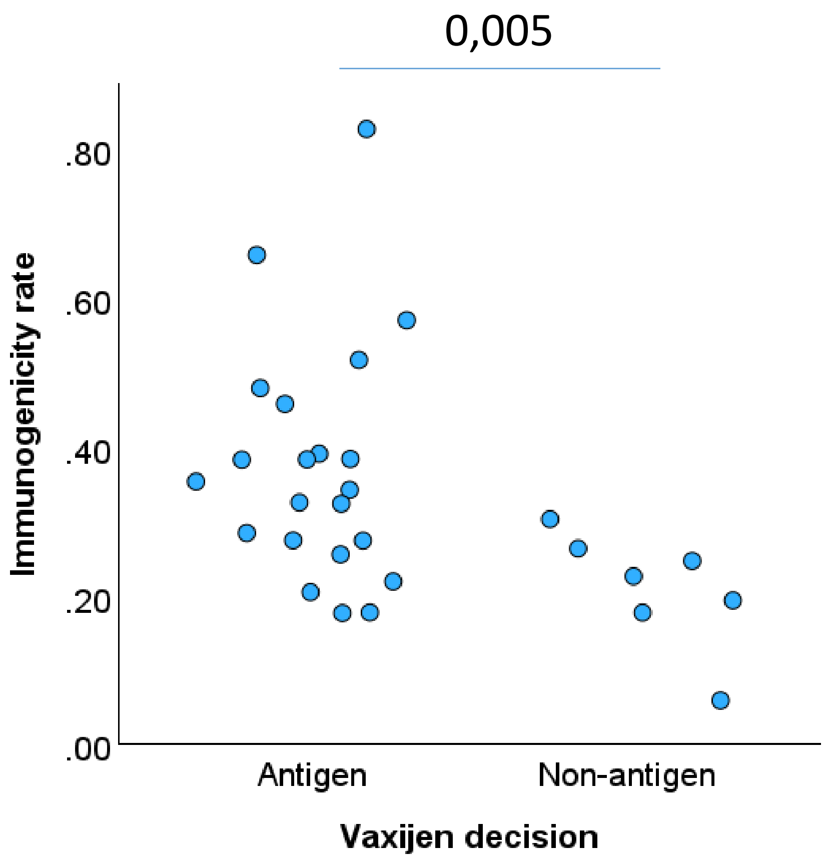

Supplementary Figure S4. Association between Immunogenicity rate and bioinformatic algorithm scores. **A**) Spearman correlation of Immunogenicity rate with netMHCpan 4.1b % Rank, netCTLpan % Rank and Syfpeithi score. **B**) Scatter plot and U-Mann test of Vaxijen decision group comparison (the *p*-value is calculated using the Mann-Whitney U test for independent variables).

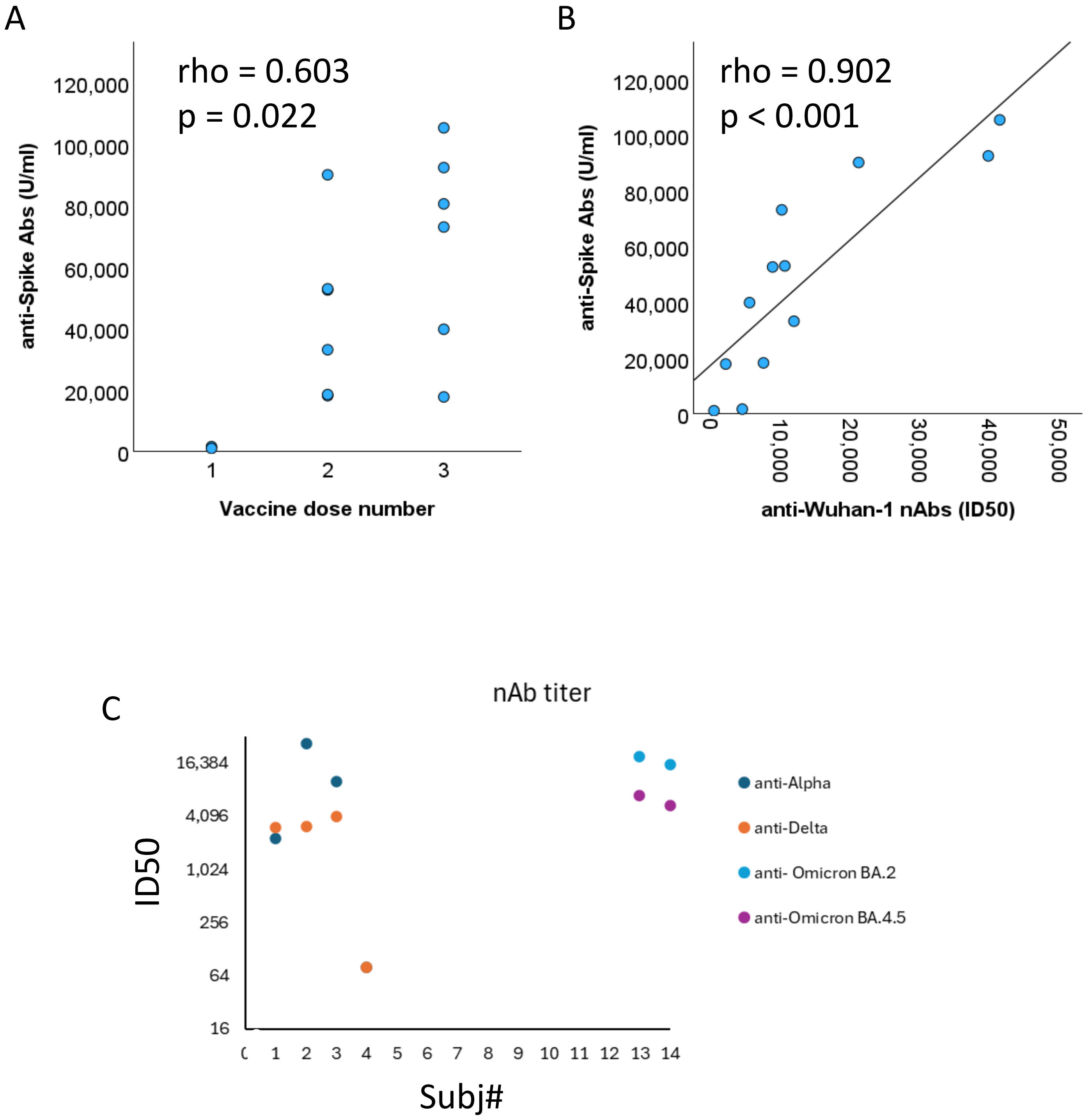

Supplementary Figure S5. B-cell response details. A) Correlation between anti-Spike Abs and vaccine dose number. **B**) Correlation between anti-Spike Abs and anti-Wuhan-1 nAbs. **C**) Antibody titer of anti-Alpha, -Delta, -Omicron BA.2 and BA.4.5 nAbs of selected subjects. Rho was calculated using Spearman's non-parametric test.

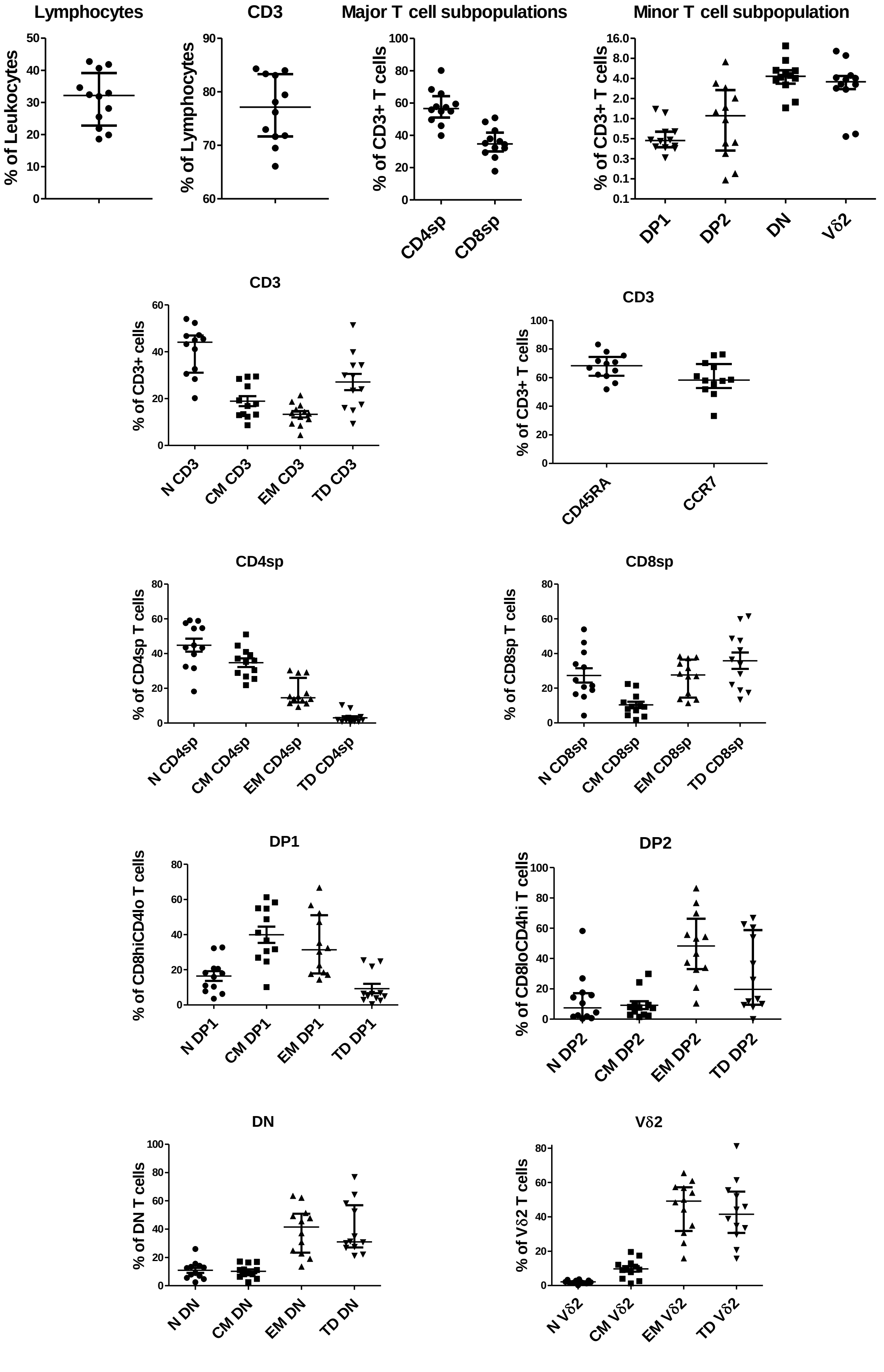

Supplementary Figure S6. Distribution of major lymphocyte subpopulation and memory subsets within the various subpopulations of T lymphocyte cells. Scatter plots show median and interquartile range.

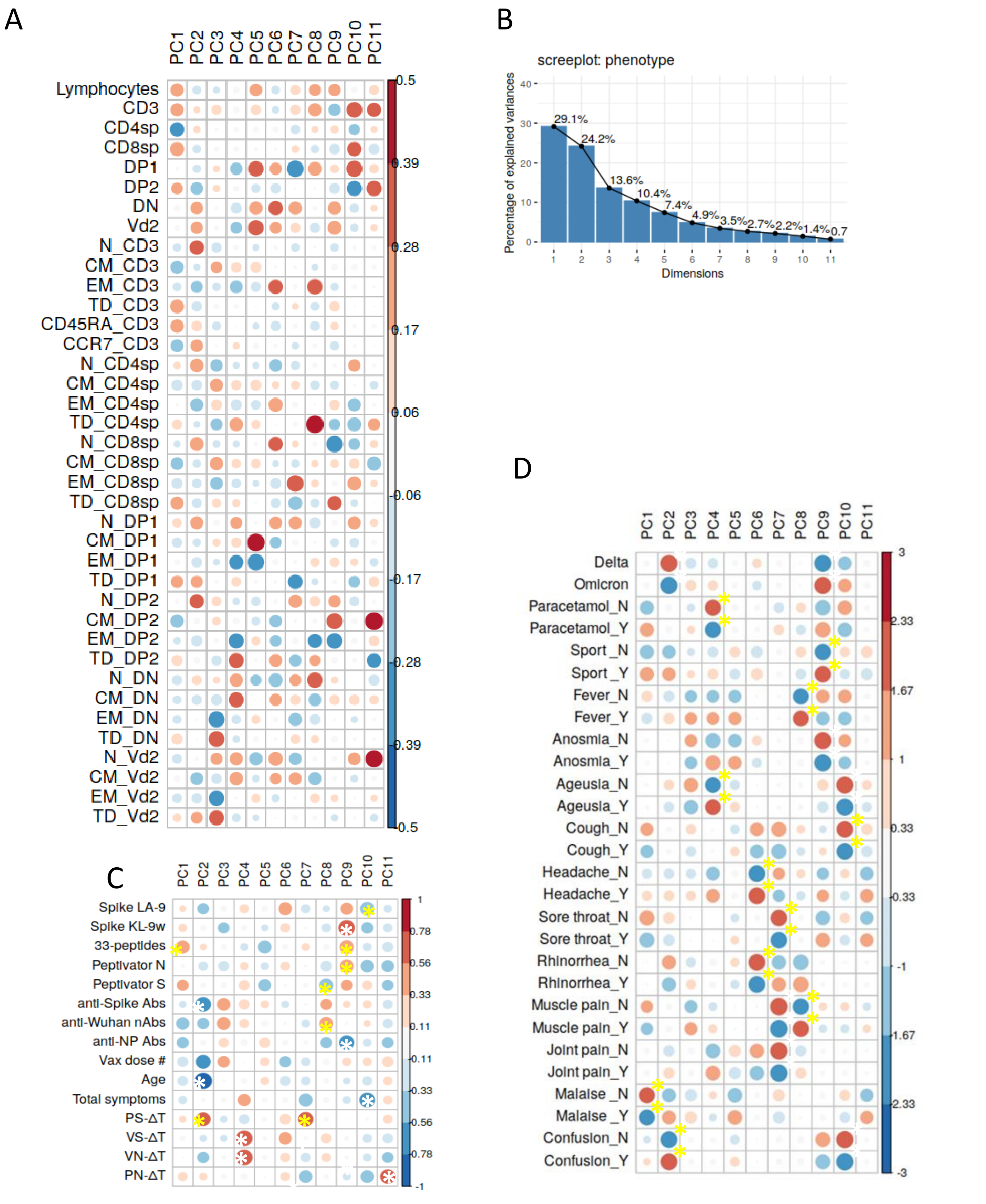

Supplementary Figure S7. Naive/memory T cell PCA. **A**) Loadings, **B**) screeplot: variance explained by each PC (Dim.s), **C**) correlation plot between quantitavive supplementary variables and PCs (Dim.s), **D**) correlation plot between qualitative supplementary variables and PCs (Dim.s) , *: p<=0,05; *: 0,05<p<0,10
